# Atmospheric Dust

**Published:** 1881-01

**Authors:** 


### ATMOSPHERIC DUST.

Every one is aware that the atmosphere holds quantities of dust in suspension. The dust betrays its presence by settling upon our clothes, furniture, and other objects ; but, on account of the minuteness of its particles, it cannot be seen as it floats in the air, except under the illumination of a strong light, as in the case of a sunbeam shining into a dark room. Besides the grains of dust which may be seen in this manner, there are others that can be perceived only through the microscope, and others smaller still, little nothings like nebulosities in the sky, which seem to become more numerous as they are sought for with more powerful instruments. These bits of dust, lifted up and carried hither and thither by the atmospheric currents, must not be overlooked—for they play a part of considerable importance in terrestrial economy, and give rise to real geological formations. Clouds of impalable dust, falling from the air in showers of considerable abundance, are not uncommon in some countries, and have been noticed in periods of history. Showers of dust, both wet and dry, are quite frequent in the Cape de Verd Islands, and are called " red fogs " by the sailors. They are also common in Sicily and Italy, and occur so often in some parts of China as hardly to attract remark. A shower of very fine dust which fell in Southern France in October, 1846, was found by the analysis of M. Dumas and the microscopic tests applied by M. Ehrenburg, to be composed of the fine sands of Guiana, and to contain the characteristic diatoms and microscopic shells of South America.

Circumstantial evidence : " Who's that frizzly, black-haired woman talking to my husband on the ottoman ? " " She's a Mrs. Cardogan Smythe." " Indeed ! she's good at flattering people, I should say, and knows how to lay it on pretty thick." " Ah ! you infer that, no doubt, from her attitude and expression ? " " Oh, dear, no ; from my husband's."

